# Comorbidity and dementia: a scoping review of the literature

**DOI:** 10.1186/s12916-014-0192-4

**Published:** 2014-10-31

**Authors:** Frances Bunn, Anne-Marie Burn, Claire Goodman, Greta Rait, Sam Norton, Louise Robinson, Johan Schoeman, Carol Brayne

**Affiliations:** Centre for Research in Primary and Community Care, University of Hertfordshire, College Lane, Hatfield, Hertfordshire, AL10 9AB UK; Research Department of Primary Care and Population Health, UCL Medical School (Royal Free Campus), Rowland Hill Street, London, NW3 2PF UK; Department of Psychology, Institute of Psychiatry, King’s College London, Guy’s Hospital Campus, London, SE1 9RT UK; Institute for Health and Society, Newcastle University, Newcastle upon Tyne, NE2 4AX UK; South Essex Partnership Trust, Luton, UK; Department of Public Health and Primary Care, University of Cambridge, Cambridge, CB1 8RN UK

**Keywords:** Dementia, Comorbidity, Diabetes, Stroke, Visual impairment, Scoping review

## Abstract

**Background:**

Evidence suggests that amongst people with dementia there is a high prevalence of comorbid medical conditions and related complaints. The presence of dementia may complicate clinical care for other conditions and undermine a patient’s ability to manage a chronic condition. The aim of this study was to scope the extent, range and nature of research activity around dementia and comorbidity.

**Methods:**

We undertook a scoping review including all types of research relating to the prevalence of comorbidities in people with dementia; current systems, structures and other issues relating to service organisation and delivery; patient and carer experiences; and the experiences and attitudes of service providers. We searched AMED, Cochrane Library, CINAHL, PubMed, NHS Evidence, Scopus, Google Scholar (searched 2012, Pubmed updated 2013), checked reference lists and performed citation searches on PubMed and Google Scholar (ongoing to February 2014).

**Results:**

We included 54 primary studies, eight reviews and three guidelines. Much of the available literature relates to the prevalence of comorbidities in people with dementia or issues around quality of care. Less is known about service organisation and delivery or the views and experiences of people with dementia and their family carers. There is some evidence that people with dementia did not have the same access to treatment and monitoring for conditions such as visual impairment and diabetes as those with similar comorbidities but without dementia.

**Conclusions:**

The prevalence of comorbid conditions in people with dementia is high. Whilst current evidence suggests that people with dementia may have poorer access to services the reasons for this are not clear. There is a need for more research looking at the ways in which having dementia impacts on clinical care for other conditions and how the process of care and different services are adapting to the needs of people with dementia and comorbidity. People with dementia should be included in the debate about the management of comorbidities in older populations and there needs to be greater consideration given to including them in studies that focus on age-related healthcare issues.

**Electronic supplementary material:**

The online version of this article (doi:10.1186/s12916-014-0192-4) contains supplementary material, which is available to authorized users.

## Background

World-wide there are an estimated 35.6 million people with dementia. By 2050 it has been estimated that this number will rise to more than 115 million [1]. Dementia is primarily a disease of old age and it often coexists with other conditions of old age. Evidence suggests that amongst people with dementia there is a high prevalence of comorbid medical conditions and related complaints [2-5]. In addition, there is evidence to support an association between the dementia syndrome including Alzheimer’s disease and cardiovascular risk factors, such as hypertension and hypercholesterolaemia [6,7].

Comorbidity amongst people with dementia presents particular challenges for primary and secondary care. Certain comorbid medical conditions may exacerbate the progression of dementia [8]. For example, cognitive decline may be accelerated in older people with type 2 diabetes [9,10]. Moreover, the presence of dementia may adversely affect and complicate the clinical care of other conditions and be a key factor in how patients’ needs are anticipated and specialist and emergency services are used [11,12]. It may also undermine patients’ abilities to self-manage chronic conditions and engage in health maintenance activities [13]. Despite this, little is known about the effects of comorbidity on processes and quality of care, patient experience, or how services are adapting to address the particular needs of this population [14].

A review of qualitative research on the experience of diagnosis and treatment of dementia [15] found very little evidence relating to the experiences of people diagnosed with dementia who have an accompanying comorbid condition. There is also a lack of research on patients’ views on the ways in which multiple conditions affect their health, well-being and clinical care [16]. The aim of this review was to identify the extent, range and nature of research activity relating to dementia and comorbidity, in particular the prevalence of co-morbidity amongst people with dementia, systems and structures that currently exist for the care of people with dementia who have comorbid medical conditions, and the experiences of people with dementia who have comorbid medical conditions and their family carers.

## Methods

The scoping review was guided by Arksey and O’Malley’s methodological framework [17,18] which includes: identifying the research question; searching for relevant studies; selecting studies; charting the data and collating and summarising and reporting the results. This approach allowed us to incorporate a range of study designs and address questions beyond those related to treatment efficacy. Whilst the scoping review has a number of similarities to a systematic review it does not typically involve quality assessment and findings are reported in a narrative format [19,20]. The inclusion criteria and methods for the review were pre-specified in a protocol [21]. The protocol was externally peer reviewed as part of the National Institute for Health Research (NIHR) funding process.

### Identifying the research question

#### Inclusion criteria

We included studies involving people with dementia who had an additional comorbid health condition. Although we included all types of comorbidities there was a particular focus on three exemplar comorbid medical conditions; diabetes, stroke and visual impairment. These three conditions were chosen because they are common in older people, generally involve some form of external monitoring and require collaboration between primary and secondary care. Moreover, they may exacerbate or influence the progression of dementia and management of these conditions; in particular, self-management is likely to be complicated by the presence of dementia [13]. We focused on community dwelling participants and excluded studies in long term care settings. We looked for studies relating to the prevalence of comorbidities in people with dementia or cognitive impairment; current systems, structures and other issues relating to service organisation and delivery; patient and carer experiences; and the experiences and attitudes of service providers. We included all study types including systematic reviews, randomised controlled trials (RCTs), controlled studies, observational studies and qualitative studies using any recognisable qualitative methodology. In addition, we included non-research items, such as clinical guidelines. We excluded studies disseminated in languages other than English.

### Searching for relevant studies

We searched for a representative range of material which provided an overview of current knowledge and that identified some key examples of developments in the organisation and delivery of care for people with dementia and comorbid conditions. We included published and unpublished literature with no date restrictions. Studies were identified by computerised searches of AMED, Cochrane Library (including CENTRAL, CDSR, DARE, HTA), CINAHL (EBSCO Publishing) (1980 to 2012), PubMed (1950 to 2013), NHS Evidence (searched September 2012) and Scopus (1966 to 2012). The electronic search strategy was developed by an experienced information scientist with input from the project team. An example of the search query for PubMed is given in Table 1. In addition, we employed extensive lateral search techniques, such as checking reference lists, performing key word searches in Google Scholar and using the ‘cited by’ option in PubMed. We also contacted experts and those with an interest in dementia, such as the Alzheimer’s Society, the Thomas Pocklington Trust, Royal National Institute for the Blind, Diabetes UK, the Stroke Association, and the Dementia and Sight Loss Interest Group. Such lateral strategies have been shown to be particularly important for identifying non-randomised studies [22]. The original electronic database searches were conducted in September 2012, with the PubMed search updated in November 2013 and lateral searches updated in February 2014.

**Table 1:** **Search strategy used in PubMed**

**Search component**	**Search terms**
**#1 Dementia and diabetes**	((Dementia OR Alzheimer OR cognitive impairment OR delirium) AND (Diabetes OR blood glucose self-monitoring) (Self management OR Self Care OR Self monitoring OR Service delivery OR Service organization OR Activities of daily living OR Caregivers OR Quality Assessment OR Quality OR Quality Indicators OR Quality of life OR Disease Progression OR Behaviour OR Impact OR Geriatric Assessment OR Severity of Illness OR Nursing Assessment OR Interprofessional OR Standard of Care OR Risk Factors OR Treatment outcome OR patient Experience) AND (Humans[Mesh]))
**#2 Dementia and stroke**	(Dementia[ti] OR Alzheimer[ti]) AND (stroke OR cerebrovascular OR CVA OR cerebrovascular disorders) AND (Self management OR Self Care OR Self monitoring OR Service delivery OR Service organization OR Activities of daily living OR Caregivers OR Quality Assessment OR Quality OR Quality Indicators OR Quality of life OR Disease Progression OR Behaviour OR Impact OR Geriatric Assessment OR Severity of Illness OR Nursing Assessment OR Interprofessional OR Standard of Care OR Risk Factors OR Treatment outcome OR patient Experience) AND (Humans[Mesh]))
**#3 Dementia and visual impairment**	(Dementia OR Alzheimer OR cognitive impairment OR delirium) AND (Eye diseases OR vision disorders OR Blindness OR visually impaired OR Nystagmus OR retinopathy OR macular degeneration OR glaucoma or cataract) AND (Self management OR Self Care OR Self monitoring OR Service delivery OR Service organization OR Activities of daily living OR Caregivers OR Quality Assessment OR Quality OR Quality Indicators OR Quality of life OR Disease Progression OR Behaviour OR Impact OR Geriatric Assessment OR Severity of Illness OR Nursing Assessment OR Interprofessional OR Standard of Care OR Risk Factors OR Treatment outcome OR patient Experience)
**#4 Dementia and comorbidity**	((Dement*[ti] OR Alzheimer*[ti]) AND ((comorbidity OR co-morbidity OR comorbid OR “other medical conditions” OR “other chronic disease*” OR multimorbidity[ti] OR multi-morbidity[ti] OR multiple disease*[ti] OR multiple morbid*[ti] OR polypathology[ti] OR associated disease*[ti] OR associated disorder*[ti]) OR co-existence[ti] OR co-existing[ti] OR concomitant[ti] OR co-occurring[ti]))
**#5**	#1 OR #2 OR #3 OR #4 OR #5

### Selecting studies and charting the data

Electronic search results were downloaded into EndNote bibliographic software and, where possible, duplicates deleted. Two authors (FB and AB) independently screened titles and abstracts identified by the electronic search and applied the selection criteria to potentially relevant papers. Data were extracted by one author using a standardised checklist and checked by a second. Any disagreements were resolved by consensus or by discussion with a third author (CG). Where results of a study were reported in more than one publication, we grouped reports together and marked the publication with the most complete data as the primary reference; the other papers describing the same study were classified as associated papers. Data extracted included type of item (for example, empirical study, review, guideline), aims/research questions, methods, study focus, participants, setting and relevant outcome data (for example, rates of comorbidities, access to treatment, patient and carer views).

### Reporting the results

Studies were grouped into the following categories: 1) prevalence, 2) quality of care, 3) views and experiences (patients, carers and health care professionals) and 4) health service organisation and delivery. All data are reported in a narrative format.

## Results

### Description of studies

**Figure 1:**
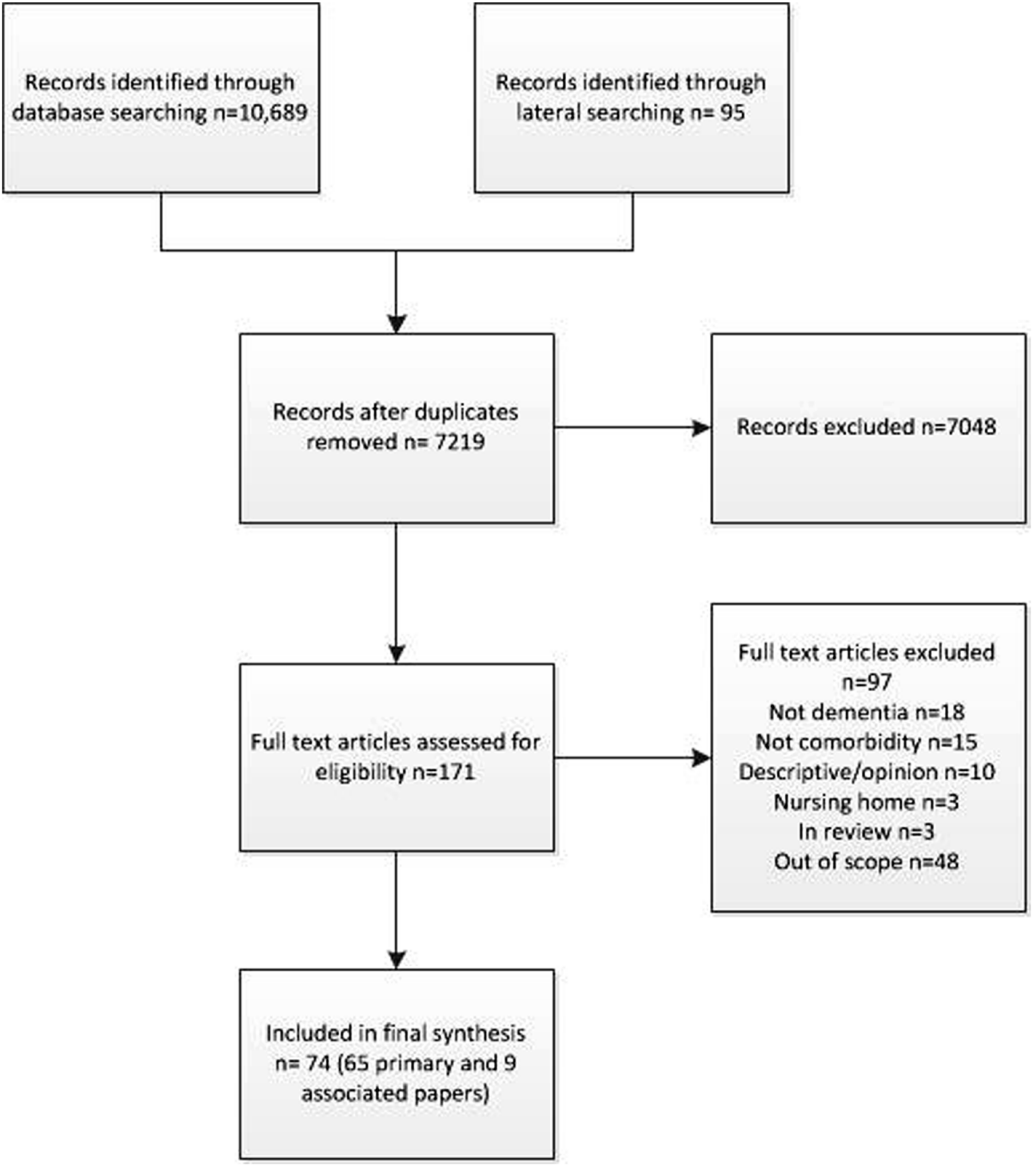
**Flow chart study selection process.**

We included 74 papers, 65 of which were classified as primary references [2,5,11,13,23-83] and nine as associated papers [84-92]. Study characteristics are summarised in the list below and details of individual studies, including the links between primary and associated papers, in Additional file 1. An overview of the selection process can be seen in Figure 1. We included studies conducted in 12 different countries but 63% were from the UK and USA; the majority were observational, qualitative or reviews. Participants were predominantly community dwelling (some had mixed samples and included those living in long-term care) and the majority were older than 70; ethnicity was not reported in 55% (n =36) of studies. In 60% of studies (n =39) participants all had dementia, in 25% (n =16) they had mixed populations including people with dementia, cognitive impairment and delirium and in 15% (n =10) they had cognitive impairment. A total of 39 studies (60%) focused on a single comorbidity and the rest on more than one comorbidity or general comorbidity/multimorbidity (for example papers relating to the experiences of people with dementia in acute hospitals). We found more evidence relating to diabetes or visual impairment than relating to stroke, and most of the evidence related to the prevalence and quality of care with less on service organisation and delivery or views and experiences of patients, carers or health care professionals.

### Overview of study characteristics

- **Study information**^**a**^
  - **Year of publication**
    - Range 1989 to 2014
  - **Country**
    - UK number = 23
    - USA number = 18
    - Europe (not UK) number = 8
    - Australia number = 3
    - Canada number = 4
    - Japan number = 2
    - International reviews/guidance number = 7
  - **Type of study**
    - Case control number = 9
    - Cohort study number = 12
    - Cross sectional number = 15
    - Guideline/policy document number = 3
    - Qualitative number = 7
    - RCT number = 3
    - Survey number = 3
    - Review/scoping number = 8
    - Other number = 5
  - **Areas covered in study**^**b**^
    - Prevalence number = 28
    - Service organisation and delivery number = 16
    - Views and experiences number = 12
    - Quality of care number = 21
  - **Setting**
    - Hospital/outpt clinic number = 30
    - Community number = 12
    - Primary care number = 7
    - Mixed community/residential care number = 5
    - Population based sample number = 8
- **Type of participants**
  - **Type of cognitive impairment**
    - Dementia number = 40
    - Cognitive impairment (including dementia and delirium) number = 8
    - Mixed population including people with dementia and MCI number = 10
    - MCI = 9
  - **Age**
    - Range 43 to 102 but majority over 70
    - Of 38 studies that gave a mean age – majority had mean age in 70s
  - **Ethnicity**
    - Not specified number = 36
    - White (or majority white) number = 14
    - Mixed number = 3
    - Mixed but majority black number = 2
    - NA (for example, review) number = 10
  - **Comorbidities included in studies**^**b**^
    - Diabetes number = 15
    - Stroke number = 3
    - Visual impairment number = 14
    - Other comorbidities number = 7 (for example, cancer, MI)
    - General comorbidity number = 12
    - More than one comorbidity (but includes one of our target conditions) number = 14
  - **Type of dementia**
    - Alzheimers number = 8
    - Mixture of different types number = 4
    - Not reported in rest

### Prevalence

Our main aim was to look at the prevalence of comorbidity (in particular stroke, diabetes and visual impairment) in people with dementia. Thirteen studies provided data about the prevalence of comorbidities in people with dementia [2,5,26,46,55,56,58,64,65,75,80-82] and two the prevalence of comorbidities in people with mild cognitive impairment (MCI) [36,72] (see Table 2). The representativeness of the samples varied. Four studies included population based samples [36,55,56,72], six recruited populations from primary care databases [2,5,26,58,64,82] and five used samples from hospitals or out-patient clinics [46,65,75,80,81] [see Additional file 2]. Data were collected from medical records in six studies [26,46,64,75,80,82], from clinical examination or interviews in six [2,36,55,56,65,72] and from a mixture of medical records and clinical examination in three [5,58,81]. The presence of dementia and comorbid medical conditions was assessed using a variety of measures [see Additional file 3].

**Table 2:** **Prevalence of diabetes, visual impairment and stroke in people with dementia**

**Study and country**	**Study type**	**Dementia/CI**	**Control/comparison**	**Eligibility criteria**	**Recruited from**	**Number whole sample**	**Number dementia/CI**	**Diabetes (%)**	**Stroke (%)**	**VI (%)**	**Notes**
Barnett [26] UK (Scotland)	Cross-sectional	Dementia	NA	Alive, permanently registered with a participating practice	Primary care –national dataset	1751841	11139	13.3	18.8	3.8	VI is blindness/low vision
Doraiswamy [2]	Cross-sectional	Dementia	NA	Diagnosis of Alzheimer’s disease, 50 or over	Community health care sites	679	679	-	-	-	61% had three or more comorbidities, 30% had vascular or heart disease. Sample included mixture of community dwelling and long-term care
Feil [36] USA	Longitudinal cross sectional	CI	No CI	Geographically defined, 65 and over	Population derived sample	7482	1774	26	34	-	
Heun [46] UK	Retrospective case control	Dementia	No dementia	Diagnosis of AD, 70+, in-patient care for at least 24 hours	Hospital in-patients	72878	634	6	3	1	VI is glaucoma. Diagnosis of diabetes less common in those with AD than controls RR 0.7 (95% CI 0.5 to 0.9). No significant difference in prevalence of ischemic stroke RR 1.3 95% CI 0.9 to 2.0, or glaucoma RR 2.0 (1.0 to 4.3)
Jara [82] UK	Retrospective cohort	Dementia	No dementia	64 and over, at least 24 months continuous enrolment, no cataract diagnosis at baseline	Primary care –national dataset	650325	8124	-	-	-	Lower rate of any cataract in AD group compared to controls HR 0.52 (95% CI 0.47 to 0.58)
Löppönen [55] Finland	Cross-sectional	Dementia	NA	Geographically defined, 65 and over	Population based	1260	112	16	24	29	PWD less likely to be diagnosed with glaucoma OR 0.36; 95% CI 0.15 to 0.86), no difference in rates of cataract p =0.287
Lyketsos [56] USA	Case–control	Dementia/CI	No dementia/CI	Geographically defined, 65 and over	Population based	695	374	20	16	-	Stroke more common in people with dementia p <0.001
McCormick [58] USA	Case–control	Dementia/CI	No dementia/CI	Aged 60 and over, members of HMO, geographically defined	Population derived (from HMO database)	154	154	6	3^a^	10	Visual problems less common in PWD (10% versus 24%)
Rait [64] UK	Cohort	Dementia	No dementia	60 and over with first code for dementia during study period, at least six months data	Primary care – national dataset	135174	22529	14	29^a^	-	No difference in prevalence of diabetes (13.9% versus 14.5%) but cerebrovascular disease more common in people with dementia (29.3% versus 13.3%)
Sakurai [65] Japan	Cross sectional	Dementia	NA	Dementia or MCI	Memory clinic	160	160	19	-	-	Dementia and CI
Schubert [5] USA	Cross-sectional	Dementia	No dementia	65 and older, seen primary care physician within two years. Excluded nursing home residents, non-English speaking	Primary care	3013	107	39	10	-	No significant difference in prevalence of DM (*P* =0.19) or stroke (*P* =0.89) between those with and without dementia
Stephan [72] UK	Cross-sectional	MCI	No MCI	65 and over	Population based	13004	1486	7	19	-	
Uhlmann[75] USA	Case control	Dementia	No dementia	65 and over, English speaking, eighth-grade or higher education, ability to complete audiometric evaluation	Adult medicine clinic	174	87	not given	not given	-	Prevalence of VI significantly higher in cases than controls (OR 2 95% CI 1.2-3.4).
Zamrini [80] USA	Case control	Dementia	NA	Probable AD, black or white (white participants matched non randomly to black participants)	Memory clinic database	334	334	18	9	10	Includes all eye diseases
Zekry [81] Switzerland	Cohort	Dementia	No dementia	75 and over. Excluded: terminal illness, disorders interfering with psychometric assessment	Hospital in-patients	349	188	19	22	-	MCI + dementia

^a^Cerebrovascular disease; − = not given. CI, cognitive impairment; MCI, mild cognitive impairment; PWD, people with dementia; VI, visual impairment.

We also included a further eight studies that reported the prevalence of dementia in people with one of our three target comorbidities [30,38,39,47,66,78,79,83] (see Table 3). Three studies included samples from out-patient clinics [38,78,79], one data from an RCT in primary care [47], two research administration or health claims databases in the USA [39] and Australia [83], one from a stroke register in Canada [66] and one a population derived community-based sample in Australia [30]. Three collected data from medical or database records [39,66,83] and the rest from face to face interviews or clinical examinations.

**Table 3:** **Prevalence of dementia in people with stroke, diabetes and visual impairment**

**Study and country**	**Type of study**	**Type of population**	**Control/comparison**	**Eligibility criteria**	**Recruited from**	**Number whole sample**	**Number dementia/CI**
Bruce [30] Australia	Longitudinal cross sectional	DM	NA	Defined by post-code, 70 years or older, diabetes	Patients living in catchment area of hospital (63% of eligible patients recruited)	223	34 (15.3%)
Feil [38] USA	Longitudinal cross-sectional	DM	NA	Diagnosis of type 2 diabetes, 60 years or older	Geriatric clinic	51	23 (45% CI)
Feil [39] USA	Cross-sectional	DM	NA	Veterans aged 65 and older with diabetes mellitus	Research administration database (Veterans Health Administration)	497000	65107 (13% dementia/CI)
Hewitt [47] UK	Questionnaire	DM	NA	Type 2 diabetes, aged 75 and older, not resident in nursing homes	Data from RCT in Primary Care	1047	235 (22.5% dementia/CI)
Zhang [83] Australia	Retrospective cohort study	DM	NA	Veterans, 65 and older, received prescription for diabetes in previous six months	Health claims database	17095	4.4%
Saposnik [66] Canada	Retrospective cohort study	Stroke and dementia	Stroke no dementia	18 years and older, first ischemic stroke	Stroke register (included patients admitted to 12 regional stroke centres in Ontario)	10658	966 (9.1%)
Whitson [78] USA	Cross-sectional	VI (macular disease)	NA	65 and older, macular disease diagnoses	Low Vision Rehabilitation clinic	101	19 (19%)
Yochim [79]	Case series	VI (glaucoma)	NA	50 and older, diagnosis of glaucoma	Glaucoma clinic	41	44% (MCI)

CI, Cognitive impairment; DM, diabetes mellitus MCI, mild cognitive impairment; RCT, randomised controlled trial; VI, visual impairment.

### Prevalence of target comorbidities in people with dementia

#### Diabetes

Twelve studies reported the prevalence of diabetes in populations of people with dementia [5,26,46,55,56,58,64,65,80,81] or MCI [36,72]. Prevalence rates varied from 6% to 39%. The two largest studies which both involved participants from national primary care data sets in the UK, one from Scotland [26] and one from the UK (primarily England) [64], reported similar prevalence rates of 13% [26] (personal communication B Guthrie) and 14% [64]. Five studies compared rates of diabetes in those with and without dementia. Of those, three studies with samples recruited from primary care databases, two from the USA and one from the UK, found similar rates between groups [5,58,64] and a study in hospital inpatients in Switzerland found no significant differences in rates of diabetes mellitus between those with and without dementia [81]. In contrast, a study of hospital in-patients in the UK found significantly fewer people with dementia diagnosed with type-2 dementia than controls without dementia [46].

#### Stroke

Nine studies reported the prevalence of stroke in people with dementia [5,26,46,55,56,58,64,80,81] and two in people with cognitive impairment [36,72]. Prevalence rates varied from 3% in hospitalised older people in the UK [46] to 34% in a sample of urban and rural community dwelling people with cognitive impairment in the USA [36]. Two studies used records from large primary care databases in the UK, one reported that rates of stroke in people with dementia were 19% [26] and the other that rates of cerebrovascular disease (including stroke) were 29% [64]. Five studies compared rates of stroke in people with and without dementia. Three found no significant difference in the prevalence of stroke [5,46,81], but one study of hospital inpatients found that stroke was more common in people with dementia [56] and a primary care based study in the UK found a greater prevalence of cerebrovascular disease in people with dementia [64].

#### Visual impairment

Four studies reported the prevalence of some form of visual impairment in people with dementia, including all eye diseases [80], glaucoma [46,55] and cataracts [55,82]. Differences in the populations studied and the way cases were identified make comparisons across studies difficult. Two studies compared rates of glaucoma in people with dementia and those without; prevalence in people with dementia was lower in a population sample in Finland [55] but not different in hospital inpatients in a retrospective case control study in the UK [46]. Two studies compared rates of cataracts. A large primary care cohort study in the UK found a lower incidence rate for cataracts in people with AD than controls [82] but a smaller population-based study in Finland reported no difference in the rates of cataracts [55].

### Prevalence of dementia in people with stroke, diabetes and visual impairment

Five studies looked at the prevalence of dementia or cognitive impairment in populations of people with diabetes. In a large population-based study in the USA, 13% of people with diabetes had dementia or cognitive impairment [39] compared with 23% in a sample recruited through primary care in the UK [47]. Two studies reported the prevalence of dementia in people with visual impairment recruited via eye clinics. In one [78] 19% of people with macular disease had dementia and in the other [79] 20% of people with glaucoma had memory impairment and 22% impaired executive functioning. One study looked at the prevalence of dementia amongst people who had had a stroke. Of more than 10,000 people on a stroke register 9% were reported to have dementia.

### Quality of care

We categorised 23 studies as relating to quality of care in some way, 11 of which compared access to treatment or receipt of services in groups with and without dementia. Ten of the 11 studies found some evidence that people with dementia were less likely to receive the same quality of care or access to services compared to those without dementia. For example, studies found that people with dementia were less likely to receive monitoring for diabetes related problems [32,74] and had reduced access to treatment, such as intravenous thrombolysis for stroke [66], surgery for cataracts [45], treatment for age-related macular degeneration (AMD) [11,33] or services for diabetes [77,83]. More details can be seen in Table 4. A German study reported that older people with a greater number of comorbidities were less likely to receive cholinesterase inhibitors for dementia [48] and a Canadian study found evidence that pain is undertreated in people with dementia and arthritis [25].

**Table 4:** **Impact of dementia and medical comorbidities on quality of care and access to treatment**

**Study ID**	**Country**	**Comorbidity**	**Study type**	**Number of participants**	**Aspect of quality of care**	**Evidence that care is different?**	**Reported differences in care/treatment**
Connolly [92]	UK	DM, Stroke	Cross sectional	700 PWD (compared to people without dementia on QOF register)	Monitoring and treatment	Yes	PWD -significantly lower on 73% of QOF indicators; including peripheral pulses check, neuropathy testing, cholesterol measures for stroke.
Curtis [33]	USA	VI (AMD)	Retrospective cohort	284380	Treatment	Yes	PWD significantly less likely to receive anti-VEGF RR 0.88 (95% CI 0.88 to 0.89)
Guijarro [45]	Spain	VI, general	Cohort	40482	Treatment	Yes	PWD had some procedures less frequently than those without dementia. For example,cataract surgery (*P* <0.001), hernia repair, orthopaedic surgery
Keenan [11]	UK	VI (AMD)	Cohort	65894 (AMD cohort, 168092 dementia cohort)	Treatment	Yes	PWD significant decrease in likelihood of hospital admission for AMD *P* <0.001
Löppönen [55]	Finland	VI, general	Cross sectional (survey)	1260 older people (112 PWD)	Diagnosis and treatment	Yes	PWD -more undiagnosed diseases compared to those without dementia (*P* =0.041) , less likely to be diagnosed with glaucoma (*P* =0.022)
Müther [60]	Germany	DM, hypertension	Retrospective matched control	216 PWD, 216 matched controls	Treatment	No	No significant differences in treatment for those with and without dementia. PWD more likely not to receive medication for hypertension or be treated with low-priced medications (not significant)
Saposnik [66]	Canada	Stroke	Cohort	877 with pre-existing dementia	Treatment	Yes	Patients with pre-existing dementia less likely to receive intravenous thrombolysis.
				877 controls (no pre-existing dementia)			
Sloan [69]	USA	Acute MI	Cross sectional	5851 admitted for AMI with dementia, 123241 admitted for AMI without dementia	Treatment	Yes	PWD less likely to have a range of invasive procedures compared to those without a history of dementia
Thorpe [74]	USA	DM, VI	Cohort	288805 (44717 PWD)	Monitoring	Yes	PWD less likely to receive HbA1c tests (73% versus 81%), LDL-C tests (61% versus 79%), and eye examinations (52% versus 63%).
Vitry [77]	Australia	DM	Cohort	20134 veterans with diabetes (includes people with dementia/CI but numbers not clear)	Treatment	Yes	Presence of dementia associated with decreased likelihood of treatment intensification (for example, addition of antidiabetic medicine or switch to insulin/different medication)
Zhang [83]	Australia	DM, VI	Cohort	17095 veterans with and without diabetes (4.4% on dementia medication)	Treatment, access to services	Yes	Patients receiving medications prescribed for dementia less likely to use diabetic and optometry⁄ophthalmology services.

AMD, age-related macular degeneration; AMI, acute myocardial infarction; CI, cognitive impairment; DM, diabetes mellitus; HbA1c, glycosylated hemoglobin; LDL-C, low-density lipoprotein cholesterol PWD, people with dementia; QOF, quality of life; VEGF, vascular endothelial growth factor; VI, visual impairment.

In addition, there was evidence that having dementia or cognitive impairment impacts on a person’s ability to undertake self-care management. For example, people with dementia and diabetes had problems understanding their condition, managing medication and monitoring their blood glucose [13,29,37,38,47].

### Views and experiences

We included 11 studies looking at views and experiences of people with dementia, their family carers and health care professionals. Six were qualitative [27,37,54,62,70], one was a mixed study including a review and qualitative study [42], two were reviews [28,34] and two were questionnaire studies [29,47]. Three studies focused on people with dementia and diabetes [29,37,47], two visual impairment [28,54], one deafness [24], one cancer [27] and four on the needs of people with dementia in general hospitals [34,42,62,70].

Literature on the experiences of older people with dementia in acute general hospitals has highlighted shortcomings in the care provided, attitudes and training of staff, the physical environment and problems with care cultures [34,42,62]. Poor communication is a major barrier to the provision of good care for people with dementia and a comorbid health condition [34,42,54]. Practitioners reported that they found it difficult to communicate with people with dementia and communication difficulties are compounded when they have additional comorbidities which make communication difficult, such as hearing loss or sight loss [24,27,54]. There were also problems with communication between different professionals and specialist teams with a lack of coordinated working between practitioners in different specialities [54]. A lack of appropriate knowledge and training was also a major barrier, with those working in acute care hospitals [42], palliative care [27] and diabetes [39] lacking knowledge about dementia. Conversely, those in dementia services may lack awareness about how to support people with dementia and visual impairment [54,88].

A qualitative study involving the views of 21 caregivers of people with dementia and type 2 diabetes found that behavioural and psychological symptoms of dementia disrupted diabetes care. In addition, carers felt that they received inadequate support in planning their relatives care, that their contribution to managing their relatives care was not always recognised and that they were often excluded from decision making [37].

### Service organisation and delivery

#### Guidelines and care pathways

Guidance on the care of older people with diabetes highlights the need to balance the benefits of diabetes treatment while minimising the risk in people with dementia [49] as this group may be at increased risk of hypoglycaemia [49,68] and guidance from the College of Optometrists states that when examining patients with dementia optometrists have a duty to carry out whatever tests are necessary to determine the patients’ need for vision care [31]. However, most guidelines are condition specific and generally fail to take into account multimorbidity or the needs of people with dementia [38,47,72,77,84,88].

A recent review identified 16 guidelines/position statements or standards (from the UK, USA and Australia) for the care of people with dementia but these generally focused on standards for providing optimal care for older people with cognitive impairment in acute hospitals or specific issues, such as hydration, nutrition or wandering [23]. They did not cover issues relating to the care of people with dementia and specific medical conditions (such as diabetes). Moreover, most models of practice for work with people with dementia do not mention visual impairment [88].

#### Models of care for older people with cognitive impairment

A number of initiatives have been developed to improve the care of older people with dementia in acute hospitals, including liaison psychiatric services [50,73] or specialist units that combine medical and mental health care for older people [41,44]. Much of the work in this area is descriptive [41] although a specialist medical and mental health unit for people with dementia has recently been evaluated in an RCT [44]. They found no difference between the specialist unit and standard care on general medical wards for their primary outcome (days spent at home) but there were significant differences in process items in favour of the intervention, and family carers were more satisfied [44]. However, the study excluded patients with clinical needs for specialist services, such as surgery or stroke units.

A scoping review of interventions for cognitively impaired older people in emergency departments found no evaluations of organisational or system level interventions and little evidence of appropriate interventions [61]. We found no evaluations of interventions aimed specifically at our three target comorbidities, although a retrospective analysis of stroke patients suggested that cognitively impaired patients benefit from admission to an acute rehabilitation stroke unit [63].

## Discussion

We included 54 primary studies, eight reviews and two guidelines that addressed issues around dementia and comorbidity. We found evidence of a lack of continuity in health care systems and structures for people with dementia and comorbidity, with little integration or communication between different teams and specialities [54]. Moreover, many models of care are focused on single diseases and do not take into account the needs of those with multimorbidity [26,84] or of their family carers. We found a number of studies reporting prevalence for our three target comorbidities. Whilst heterogeneity in the populations and differences in the way that conditions were ascertained make comparisons across studies difficult the data do suggest that rates of diabetes in people with dementia may be between 13 and 20 percent and of stroke between 16 and 29 percent.

We undertook a scoping review rather than a systematic review. A limitation of such an approach is that the review does not include an assessment of the quality of the included studies or evaluate the effectiveness of interventions. However, evaluative research in this area is limited and our aim was to examine the extent, range and nature of research activity around patient and carer need, health care provision and service organisation for people with dementia and comorbidity. Moreover, whilst we did not undertake formal quality assessment we did extract methodological information on study populations and data collection methods that aided in the interpretation of the evidence. We set out to include a representative rather than exhaustive range of literature and it is possible that we have missed relevant studies or guidelines. However, our search strategy was guided by systematic review methodology and we employed extensive database and lateral searches. We are confident, therefore, that this scoping review provides a comprehensive summary of current evidence relating to dementia and comorbidity for people living in the community, particularly in relation to diabetes, stroke and visual impairment. We did, however, exclude studies in care homes where there are likely to be considerable levels of dementia and comorbidity. Moreover, the majority of our studies focus on single comorbidities and the review does not cover the presence of multiple conditions. The support that people with dementia and multiple health conditions need may be different than those with less complex needs. Further studies are needed to understand the complexity within multiple comorbidities.

To our knowledge, this is the first review of research and practice on co-morbidity in dementia. Most research on multimorbidity has been concerned with its effect on physical functioning and its measurement, with little research investigating the effect on processes of care or what constitutes ‘best care’ for these patients [14]. Moreover, qualitative research on dementia has tended to focus on the experience of living with dementia as a single disease [15]. This review sets out current research and knowledge about the impact of comorbidities on people diagnosed with dementia and what is known about their experiences of health care.

This review suggests that significant numbers of people with dementia have a comorbid health condition, such as diabetes or stroke. This has serious implications for the way that specialist services for conditions such as diabetes are delivered for people with dementia. Despite these high levels of comorbidity in people with dementia there was evidence that they did not have the same access to treatment and monitoring for conditions such as visual impairment and diabetes as those with similar comorbidities but without dementia. There may be a variety of factors that contribute to this finding. For example, people with dementia may be less likely to attend regular appointments or to notice or report relevant symptoms and they may be more reliant on carers to manage and facilitate appointments [11]. It is also possible that clinicians may be more reluctant to investigate and treat patients with dementia either because of the difficulties involved in securing patient cooperation or because treatments are considered inappropriate for older patients with multimorbidity. In addition, if the dementia is symptomatic (such as the behavioural and psychological symptoms associated with dementia) then dementia may become clinically dominant and detract from the management of conditions such as diabetes mellitus [93,94].

Much of the available literature relates to the prevalence of comorbidities in people with dementia or issues around quality of care. There is less evidence relating to service organisation and delivery, patient and carer preferences or the decision making processes of clinicians involved in their care. Whilst there is literature relating to models of care for older people with cognitive impairment, this is focused on in-patient care for patients who would otherwise be admitted to a general medical ward and does not provide guidance on the management of specific conditions (such as diabetes or stroke) in people with dementia. In addition there is little guidance on the care of people with dementia and long-term conditions living in the community. Family caregivers of people who have dementia and other health conditions face great challenges in managing both conditions and dealing with the impact of accompanying behavioural and psychological symptoms of dementia on care routines [37]. Despite this, little is known about how family carers can best be supported, or how health care services should adapt to address the particular needs of this population.

### Recommendations for research

The review highlights significant gaps in the evidence. There is currently a lack of research on the experiences of patients and their family carers on living with dementia and comorbid conditions. Even less is known about healthcare providers experiences of managing people living with dementia and comorbid health conditions and how the presence of dementia influences the care they receive for their comorbidity. These are areas that are currently being explored in an NIHR study on dementia and comorbidity [21]. In addition, there is a need for more detailed epidemiological work on the prevalence of comorbidities in people with dementia and the appropriateness of treatment and referral for this group. People with dementia should be included in debate about the management of comorbidities in older populations, and there needs to be greater consideration given to including them in studies and trials that focus on age-related healthcare issues. Equally, dementia-specific studies need to consider the impact of comorbidities on the experience of living and dying with dementia.

## Conclusions

The prevalence of comorbid conditions in people with dementia is high. Whilst current evidence suggests that people with dementia may have poorer access to services the reasons for this are not clear. There is a need for more research looking at the ways in which having dementia impacts on clinical care for other conditions, how process of care and different services can adapt to the needs of people with dementia and comorbidity, and what interventions might improve access to services and the physical health of people with dementia. Clinical guidance should consider the particular needs of those with dementia and comorbid health conditions.

## Endnotes

^a^All numbers refer to the numbers of studies not individual participants.

^b^some studies classified in more than one category.

## Additional files

Additional file 1:
**Table of included studies.**
Additional file 2:
**Prevalence studies – information on study populations, recruitment and participation.**
Additional file 3:
**Prevalence studies – information on methods of data collection.**

## Abbreviations

AMD: age-related macular degeneration
AMED: Allied and Complementary Medicine Database
CENTRAL: Cochrane Central Register of Controlled Trials
CDSR: Cochrane Database of Systematic Reviews
CI: cognitive impairment
DARE: Database of Abstracts of Reviews of Effectiveness
HTA: health technology assessment
MCI: mild cognitive impairment
RCT: randomised controlled trial
VI: visual impairment
