# Supplementary material for: Comorbidity and dementia: a scoping review of the literature
Source: BMC Med. 2014 Oct 31;12:192. doi: 10.1186/s12916-014-0192-4 (PMC4229610; doi:10.1186/s12916-014-0192-4)
Supplement: Additional file 1: — Table of included studies. [file 12916_2014_192_MOESM1_ESM.docx]

**Details of Included Studies**

| **Study ID** | **Country** | **Study design** | **Number of Participants** | **Aims/research questions** | **Study focus^1^** | **Comorbidity** | **Age^2^** | **sex (% female)** |
| --- | --- | --- | --- | --- | --- | --- | --- | --- |
| ACSQHC 2013 | Australia | Rapid review | NA | To identify best practice in caring for patients with cognitive impairment in acute hospital settings | s | General | NA | NA |
| Allen 2005 (Allen 2006) | UK | Qualitative | Total: 43  12 service users with hearing impairment  16 practitioners  8 relatives  7 BSL interpreters | Consultation exercise looking at issues for people with dementia with hearing loss | V | Hearing impairment | Not reported | Not reported |
| Balfour 2003 | Canada | Cross sectional | Total: 460 PWDs  With arthritis =245  No arthritis =215 | Are patients with AD inappropriately prescribed neuroleptics and benzodiazepines | Q | Arthritis | 84.33 | 73% |
| Barnett 2012 (Guthrie 2012) | UK | Cross sectional | 1,751,841 patients from database of primary care practices | Examined distribution of multimorbidity and comorbidity in relation to age and socioeconomic deprivation | P | General multimorbidity but includes information on diabetes and stroke | 16.6% aged 65 and over | 50.5% |
| Bartlett 2011 (Bartlett 2009) | UK | Qualitative & scoping | 7 practitioners  4 students | To increase knowledge of visual issues and eye health for people with dementia | V | VI | Not reported | Not reported |
| Bartlett 2012 | UK | Qualitative | 5 healthcare professionals | How healthcare professionals assess the needs of and communicate with a person dying from cancer with a coincidental dementia | V | Cancer | Not reported | 100% |
| Bayer 1994 | UK | Prevalence study | 26 older people with diabetes (in all but 5, diabetes pre-dated dementia) | Impact of dementia on diabetic care | V | Diabetes | 78.5 (70-91) | 73% |
| Bayer 2002 | Germany | Cross sectional | 112 PWDs | Examine the occurrence of glaucoma among patients with AD | P | VI | 71.4 (no glaucoma group), 72.9 (glaucoma group) | 64% |
| Bruce 2003 | Australia | Cross sectional | 223 older people with diabetes | Determine whether the prevalence of dementia in older diabetics warrants an active screening approach | P | Dementia and depression in people with diabetes | 76.5 | 48.8% |
| College of Optometrists 2012 | UK | Guidelines | NA | Guidelines for Optometrists examining a patient with dementia or cognitive impairment | S | VI | NA | NA |
| Connolly 2013 (Connolly 2012) | UK | Observational, cross-sectional review of primary care records | 700 PWDs (compared to people without dementia on QOF register) | Evaluate the quality of medical care for vascular diseases provided to people with dementia | Q | Stroke and diabetes | 82.1 (range 43-102) | 66% |
| Curtis 2012 | USA | Retrospective cohort study | 284,380 (with and without dementia) | Examine use of treatments for Age-related Macular Degeneration (AMD) | Q | VI (AMD) | 81 | 64% |
| Dewing 2014 | UK | Literature review | NA | Review literature on the acute care of PWD in general hospitals | V, S | General | NA | NA |
| Doraiswamy 2002 | USA | Cross sectional | 679 older people with AD | Examine the prevalence of comorbid medical illness in AD patients | P | General | 87 | Community 55.6%, Assisted living 75.8%, Nursing home 83.5% |
| Doucet 2008 | France | Cross sectional | 238 older people with diabetes | Identify characteristics of elderly diabetic patients and evaluate relationship between glycaemic control and complications of diabetes | Q | Diabetes | 82.2 | 58% |
| Feil 2003 | USA | Longitudinal cross-sectional | 7,482 older adults | Examine relationship between CI, common chronic medical illnesses and risk of mortality in older people | P | Several but includes diabetes and stroke | 51% aged 85 and over | 61% |
| Feil 2009 | USA | Cross-sectional & longitudinal study | Total: 51 people with diabetes (27 with caregiver) | Examine role of CI and caregiver support in diabetes care adherence and glycaemic control | P, Q | Diabetes | 78 yrs (range = 62-90) | 100% Male |
| Feil 2011a | USA | Qualitative | 21 caregivers of PWD and diabetes | Explore caregivers' challenges and quality of life issues managing diabetes in PWD | V | Diabetes | 65-90 yrs | Majority female (numbers not given) |
| Feil 2011b | USA | Cross sectional database analysis | 497,000 veterans with diabetes (with & without CI/dementia) | Examine the relationship between management of diabetes mellitus and hypoglycaemia in older adults with dementia | P, Q | Diabetes | 100% >65yrs 44% > 75 yrs | 2% |
| Formiga 2013 | Spain | Prospective population based survey | 515 PWDs | Evaluate comorbidity in elderly with dementia to determine differences according to dementia severity | P | General | 81 | 70% |
| George 2011 | UK | Systematic review |  | Examine literature to determine the evidence for the effectiveness of joint geriatric/psychiatric wards | S | General | NA | NA |
| Gladman 2012 | UK | Qualitative study (and review) | 60 HCPs, 36 pt & carer interviews | Elicit staff and organisational attitudes to dealing with older patients with CI, understand impact on patients, carers and staff, identify potential improvements | V | General | Patients 86.8 (70-99), Carers 63 (46-79) | 80% HCPs, 56% patients |
| Gold 1996 | USA | Case Control study | 52 pts attending memory clinic (30 dementia, 22 CI) | Determine prevalence and characteristics of hearing loss, determine whether screening tools are adequate and whether patients with AD can adequately report hearing problems | P | Hearing Loss | Not reported | 83% |
| Goldberg 2013 | UK | RCT | 310 Int grp, 290 control | Develop and evaluate a best practice model of general hospital acute medical care for older people with cognitive impairment | S | General | Median age 85 | 55% int group, 40% control |
| Guijarro 2010 | Spain | Cohort study | 40,482 PWDs | Determine prevalence and clinical characteristics of hospitalised dementia patients | P, Q | Several including cataracts and diabetes | 78 | Not reported |
| Heun 2013 | UK | Retrospective case control | 634 with AD  72244 control group | Differences between AD & Non-AD patients in terms of comorbid diseases at hospital admission and which comorbidities contribute to mortality | P | General | AD 85.1(SE 8.2), Control 80.8 (SE 7.4) | AD 65%, Control 51 |
| Hewitt 2010 | UK | Questionnaire survey | 1047 older people with Type 2 diabetes | Examine knowledge and management of diabetes in older people | P, V | Diabetes | 80.9 (range 75-100) | 53.3% |
| Hill 2013 | UK | Guidance | NA | Highlight importance of recognising relationship between diabetes and dementia, the impact one condition has on the other, and maximising the benefits and safety of diabetes treatments while minimising risks | Q | Diabetes | NA | NA |
| Hoffman 2011 | Germany | Cohort study | 1848 PWD | Determine whether comorbidity and polypharmacy influence prescription of ChEIs in PWD | Q | General comorbidity but some mention of visual disturbances | 78.7 | 47.6% |
| Holmes 2010 | UK | Literature review, mapping and case study | 10 case study sites: recruited 757 in the referred cohort and 975 in the comparison cohort | Establish what service models are being used to improve the care of older people with mental health problems in general hospitals and what impact these models might have on outcomes | S | General | 80 | Comparison 55%, referred 64% |
| Ishii 2008 | Japan | Case series | 88 people with VI | Evaluate the influence of cataract surgery on cognitive function & depressive mental status of elderly patients | Q | VI | 75.3 (range 55-93) | 64% |
| Jara 2011 | USA (but UK data) | Retrospective cohort study | 8124 AD  642,325 non-AD | Evaluate the occurrence of cataracts in people with AD compared with the general population | P | VI | 64 years + | 68% AD cohort, 54% non-AD cohort |
| Jefferis 2011 | UK | Literature review | NA | What are the implications for practice relating to benefits of cataract surgery for PWD? | Q | VI (cataracts) | NA | NA |
| Jones 2007 (Trigg 2005) | UK | Scoping review | NA | Review of research on PWD and serious sight loss | P, Q | VI | NA | NA |
| Keenan 2013 | UK | Cohort Study | 65,894 (AMD cohort); 168,092 (dementia cohort) | Are PWD more or less likely to be admitted to hospital for AMD treatment | Q | VI (AMD) | Majority over 65 | 61% |
| Lawrence 2009 (Lawrence 2008, 2010, 2009) | UK | Qualitative | 17 PWD and VI  17 family caregivers  18 care professionals | The experiences and needs of older adults with visual impairment and dementia | V | VI | Range 65-99 (18/19 aged 75 and over) | 63% |
| Löppönen 2004 | Finland | Cross sectional population based study | Total: 1260 older people 112 PWDs | Study of undiagnosed diseases in older people with and without dementia | P, Q | Several including diabetes, stroke and VI | 64 years + | 58% |
| Lyketsos 2005 | USA | Case control | 695 older people  149 PWDs  225 with CI  321 no dementia/CI | Investigated medical comorbidity in persons with dementia and cognitive impairment | P | General | Dementia 83.89, CIND 82.38, No Dem 79.93 | Dementia 64.4%, CIND 53.8%, No Dem 54.8% |
| Mackenzie 2013 | Canada | RCT | 56 stroke and MCI | Investigate whether nurse case management interventions results in lowered BP | S | Mainly stroke but includes diabetes | 59% >65 yrs | 30% |
| McCormick 1994 | USA | Case control | Total: 375 (154 with dementia, 92 CI,  129 controls) | Compare comorbidity in AD patients with non-AD patients | P | General | 76 | Dementia 68%, CI 57%, Controls 63% |
| McKeefry 2010 (McKeefry 2010) | UK | Scoping | NA | To develop guidelines for Optometrists for best practice with patients with dementia and sight loss | S | VI | NA | NA |
| Müther 2010 | Germany | Retrospective matched control study | 216 PWDs  216 matched controls without dementia | Are patients with dementia treated differently from patients without dementia | Q | Several includes diabetes | 82.7 (dementia group), 82.2 (non-dementia group) | 77.3% |
| Parke 2011 | Canada | Scoping review | Included 15 evaluation studies | Scope research on CI in older adults who visit ED of acute care hospitals and evaluation of effectiveness of programmes | S | General | Aged 65+ | NA |
| Parke 2013 | Canada | Qualitative | 10 adult-family caregiver dyads, ten ED nurses, 4 nurse practitioners | Identify factors that facilitate or impede safe transitional care in the ED for community dwelling older adults | S, V | General | PWD 83 (77-90), carer 57-81 (51-84 | Not specified |
| Rabadi 2008 | USA | Retrospective analysis | 668 (435 with CI) | Can stroke patients with CI benefit from admission to an acute rehabilitation unit? | S | Stroke | 70.3 (22-96) | 53% |
| Rait 2010 | UK | Cohort | 22529 PWDs  112645 matched non dementia  Ratio of 1:5  Dementia: Non dementia | Estimate survival after diagnosis of dementia in primary care compared with people without dementia and determine incidence of dementia | P, Q | Several including stroke & diabetes | 82.2 | Dementia 67.9%, no dementia 55.8% |
| Sakurai 2010 | Japan | Prevalence study | 113 PWDs | Investigated prevalence of coexisting diseases in PWD | P | Several including diabetes | 78.6 | 73% |
| Saposnik 2012 | Canada | Retrospective cohort study | 877 with pre-existing dementia  877 controls (no pre-existing dementia) | Determine if pre-existing dementia is an independent predictor of all-cause mortality & disability after ischemic stroke | P, Q | Stroke | 82 | 60% |
| Schubert 2006 | USA | Cross sectional | Total: 3013  107 PWDs | Compare the medical comorbidity of older patients with and without dementia in primary care | P | Several including stroke and diabetes | 73.4 | 66.6% |
| Shah 2007 | UK | Survey | 100 Optometry Practices | Investigate the accessibility of a sight test for an older person with dementia | Q | VI | NA | NA |
| Sinclair 2000 | UK | case control | 396 with diabetes 393 matched controls | Whether cognitive impairment is associated with changes in self-care behaviour and health service use in older diabetics | Q | Diabetes | 74.9 diabetics, 74.8 controls | 51% |
| Sinclair 2011 | Europe | Guidance | NA | Support clinical decisions in older people with diabetes and enhance high quality diabetes care by the use of best available evidence | S | Diabetes | NA | NA |
| Sloan 2004 | USA | Cross sectional (retrospective chart review) | 5,851 admitted for AMI with dementia  123,241 admitted for AMI without dementia | Differences in mortality after admission for acute myocardial infarction (AMI) and in treatments for AMI between patients with and without dementia | S | Acute MI | dementia 81.6, no dementia 75.5 | dementia 57.2%, no dementia 46.3% |
| Spencer 2013 | UK | Qualitative | 40 (20 from specialist unit, 20 standard care) | Examine carer's views and experiences of delivery of patient care for PWD in acute general hospital in order to evaluate specialist medical and mental health unit compared with standard hospital wards | V | General | Not reported | Not clear |
| Stenvall 2012 | Sweden | RCT | 64 patients with fractures NoF  28 Intervention  36 Controls | Whether a multidisciplinary postoperative intervention program reduced postoperative complications and improved functional recovery among people with dementia | S | Hip fracture | >70 years | Intervention 79%, control 69% |
| Stephan 2011 | UK | Cross sectional | Total: 13004  587 PWD  319 MCI  608 no cognitive impairment | Compared the pattern of disease co-morbidity across different cognitive groups and whether co-morbidity is a risk factor for dementia progression | P | Several but includes diabetes and stroke | Dementia group - with health conditions 79.8, no health conditions 81.9 | Dementia with health conditions 60.1%, no health conditions 73.2% |
| Thorpe 2012 | USA | Retrospective cohort study | 288,805  44,717 PWDs | Examined how recommended monitoring of diabetes differed for people with and without comorbid dementia | P, Q | Diabetes | 26% aged 65-69, 48% aged 70-79 25.9% aged 80+ | 60% |
| Todres 2010 | UK | Retrospective analysis | Not given | Evaluate whether implementation of Rapid Assessment, Interface and Discharge (RAID) integrated model improves access to psychiatric assessment and reduces cost of health service provision in an acute hospital | S | General | Mean age of referrals from all wards 65.7. 23% of RAID group and 20% of control group were aged over 75. | RAID group 54%, comparison group 40% |
| Uhlmann 1989 | USA | Case control | 200 people with hearing impairment 100 with dementia 100 controls | Whether hearing impairment contributes to cognitive dysfunction in older adults | P | Hearing impairment | 77 | 58% |
| Uhlmann 1991 | USA | Case control | 87 PWDs  87 matched controls | How impaired visual acuity is associated with dementia and cognitive dysfunction in older adults | P | VI | 77 | 58% |
| Vitry 2010 | Australia | Retrospective cohort study | 20,134 veterans with diabetes (includes people with dementia/CI but numbers not clear) | Whether the number of comorbid conditions unrelated to diabetes delays therapeutic progression of diabetes treatment | Q | Diabetes | 77.3 | 36% |
| Whitson 2010 | USA | Cross-sectional | 101 people with macular disease | Prevalence of comorbid cognitive impairment among older adults referred to Low Vision Rehabilitation for macular disease | P | VI (macular disease) | 80.1 | 65% |
| Yochim 2012 | USA | Case series | 41 Glaucoma patients | Prevalence of cognitive impairment, depression and anxiety in older adults with glaucoma | P | VI | 70 | 70% |
| Zamrini 2004 | USA | Case control | 999 people with AD | Prevalence of comorbid illness in black and white patients with probable AD | P | Several but includes diabetes and eye disease | Female 75.1,  Male 73.1 | 49% |
| Zekry 2008 | Switzerland | cohort | 349 inpatients 43.3% dementia  10.6% MCI | Comorbid conditions, functional and nutritional status in hospitalised patients with dementia and MCI | P | Several but includes data on stroke and diabetes | 85 | 76% |
| Zhang 2010 | Australia | Retrospective cohort study | 17 095 veterans with and without diabetes (4.4% on dementia medication) | Impact of co-morbidity on health service utilization by Australian veterans with diabetes | Q | Diabetes | 81 | 44% |

1: P= prevalence, Q = quality of care, V = views & experiences, S = service organisation and management, 2 = unless otherwise stated refers to mean age and range, PWD = people with dementia, CI = cognitive impairment, VI = visual impairment
