# Supplementary material for: Comorbidity and dementia: a scoping review of the literature
Source: BMC Med. 2014 Oct 31;12:192. doi: 10.1186/s12916-014-0192-4 (PMC4229610; doi:10.1186/s12916-014-0192-4)
Supplement: Additional file 2: — Prevalence studies – information on study populations, recruitment and participation. [file 12916_2014_192_MOESM2_ESM.docx]

**Prevalence studies- Information on study populations, recruitment and participation**

| **Study and country** | **Type of study** | **Type of population** | **Eligibility criteria defined?** | **Method of selection** | **Nature of population** | **Number**  **Participants** | **Participation rate** |
| --- | --- | --- | --- | --- | --- | --- | --- |
| Barnett 2012  UK (Scotland) | Cross-sectional | General | Alive, permanently registered with a participating practice | National dataset | Primary care (about one third of all Scottish population) | 11139 | All patients registered with primary care practice |
| Bruce 2003  Australia | Longitudinal cross sectional | DM | Defined by post-code, 70 yrs or over, diabetes | Initial 223 members of a cohort of 529 participants | Community based volunteers who consented to take part | 223 | Initially recruited 63% of those who were eligible |
| Doraiswamy 2002 | Cross-sectional | Dementia | Diagnosis of Alzheimer’s disease, 50 or over | From a variety of community health care sites | Volunteers who consented to take part | 679 | Not clear |
| Feil 2003  USA | Longitudinal cross sectional | CI | Geographically defined, 65 and over | Secondary data analysis of dataset from community based study | Community based volunteers who consented to take part | 7482 (1774 CI) | 80% (baseline interview) |
| Feil 2009  USA | Longitudinal cross-sectional | DM | Diagnosis of type 2 diabetes, 60 years or over | Electronic medical diagnosis of type 2 diabetes | Geriatric medical clinic | 51 | Not clear |
| Feil 2011b  USA | Cross-sectional | DM | Veterans aged 65 and over with diabetes mellitus | Secondary data analysis of research administration database (Veterans HA, Medicare and Medicaid) | Population based sample – large national healthcare system database | 497,000 | All patients on database |
| Heun 2013  UK | Restrospective case control | Dementia | Diagnosis of AD, 70+, in-pt care for at least 24 hours | Consecutively admitted in-patients | Hospital | 634 | All pts who met criteria were included |
| Hewitt 2010  UK | Questionnaire | DM (CI) | Type 2 diabetes, aged 75 and over, not resident in nursing homes | Secondary data analysis of intervention arm of RCT | Primary care | 1047 | Not clear |
| Jara 2011  UK | Retrospective cohort | Dementia | 64 and over, at least 24 months continuous enrolment, no cataract diagnosis at baseline | National dataset | Primary care | 650, 325 (8124 dementia) | All patients registered with primary care practice |
| Löppönen 2004  Finland | Cross-sectional | Dementia | Geographically defined, 65 and over | All those who met criteria invited in random order | Population based | 112 | 82% |
| Lyketsos 2005  USA | Case-control | Dementia/CI | Geographically defined, 65 or older | All those who met criteria invited to participate | Population based | 695 (374 with dementia) | 90% |
| McCormick 1994  USA | Case-control | Dementia/CI | Aged 60 and over, members of HMO, geographically defined | Sub sample recruited from database (not clear how chosen) | Population derived (from HMO database) | 154 | Not clear |
| Rait 2010  UK | Cohort | Dementia | 60 and over with first code for dementia during study period, at least 6 months data | National dataset (practices that met standards for acceptable levels of data recording) | Primary care | 135174 (22529 dementia) | All patients registered with primary care practice |
| Sakurai 2010  Japan | Cross sectional | Dementia | Dementia or MCI | Consecutive outpatients attending memory clinic | Memory clinic | 160 | Not clear |
| Saposnik 2012  Canada | Retrospective cohort study | Stroke & dementia | 18 yrs and over, first ischemic stroke | Stroke register | Clinical database | 10,658 | All stroke patients attending 12 regional stroke centres |
| Schubert 2006  USA | Cross-sectional | Dementia | 65 and older, seen primary care physician within 2 yrs. Excluded nursing home residents, non-English speaking | Primary care practice centres | Primary care | 3013 (107 dementia) | Not clear |
| Stephan 2011  UK | Cross-sectional | MCI | 65 and over | Randomly selected from health authority lists in 5 areas of UK | Population based | 13004 (1486 dementia) | Not clear |
| Uhlmann 1991  USA | Case control | Dementia | 65 and over, English speaking, eighth-grade or higher education, ability to complete audiometric evaluation | Computer searches of clinic records | Out-patient clinic | 174 (87 dementia) | 70% |
| Whitson 2010  USA | Cross-sectional | VI (macular disease) | 65 and over, macular disease diagnoses. | All eligible pts attending clinic invited to participate | Out-patient clinic | 101 | 74% |
| Yochim 2012 | Case series | VI (glaucoma) | 50 and over, diagnosis of glaucoma | All eligible pts attending clinic invited to participate | Out-patient clinic | 41 | Not reported |
| Zamrini 2004  USA | Case control | Dementia | Probably AD, black or white (White participants matched non randomly to black participants) | Computer searches of clinic records | Memory clinic | 334 | All those eligible during study period |
| Zekry 2008  Switzerland | Cohort | Dementia | 75 and over. Exc: terminal illness, disorders interfering with psychometric assessment. | Random sample of all pts admitted selected each day | Hospital in-patients | 349 (188 dementia) | 85% |
| Zhang 2010  Australia | Retrospective cohort study | DM | Veterans, 65 and over, received prescription for diabetes in previous 6 months | Research administration database (Dept Veterans’ Affairs) | Population derived (from database of veterans) |  | All eligible pts on database |
