# Supplementary material for: Comorbidity and dementia: a scoping review of the literature
Source: BMC Med. 2014 Oct 31;12:192. doi: 10.1186/s12916-014-0192-4 (PMC4229610; doi:10.1186/s12916-014-0192-4)
Supplement: Additional file 3: — Prevalence studies – information on methods of data collection. [file 12916_2014_192_MOESM3_ESM.docx]

**Prevalence studies - information on methods for data collection**

| **Study and country** | **Type of study** | **Type of population** | **Method of data collection** | **How dementia/CI defined/assessed?** | **How diabetes defined/ recorded?** | **How stroke defined/**  **recorded?** | **How VI defined/recorded?** | **Other** |
| --- | --- | --- | --- | --- | --- | --- | --- | --- |
| Barnett 2012  UK (Scotland) | Cross-sectional | General | Electronic medical records | Read code ever recorded | Read code ever recorded | Read code ever recorded | NA |  |
| Bruce 2003  Australia | Longitudinal cross sectional | DM | Face to face interview, clinical examination | MMSE (<24/30), IQCODE (>3.61), Cambridge exam for mental disorders of the elderly  + diagnostic exam | Clinical & biochemical assessment |  |  |  |
| Doraiswamy 2002 | Cross-sectional | Dementia | Face to face interview and medical records | Diagnostic criteria for probable AD (NINADS-ADRDA) | NA | NA | NA | The Cumulative illness rating scale-geriatric (CIRS-G) |
| Feil 2003  USA | Longitudinal cross sectional | CI | Face to face interview | Modified version of Pfeiffers short portable mental status questionnaire (SPMSQ) | Self-reported | Self-reported | NA |  |
| Feil 2009  USA | Longitudinal cross-sectional | DM | Face to face interview | Cognitive Abilities Screening Instrument (CASI) | Diagnosis confirmed by physician & patient | NA | NA |  |
| Feil 2011b  USA | Cross-sectional | DM | Medical records | ICD codes | 2 or more DM specific ICD codes (ICD9-CM) | NA | NA |  |
| Heun 2013  UK | Restrospective case control | Dementia | Retrospective data from clinical records | ICD-10 diagnostic category | Registered diagnosis in medical records | Registered diagnosis in medical notes | Registered diagnosis in medical notes |  |

| **Study and country** | **Type of study** | **Type of population** | **Method of data collection** | **How dementia defined/assessed?** | **How diabetes defined/ recorded?** | **How stroke defined/recorded?** | **How VI defined/recorded?** | **Other** |
| --- | --- | --- | --- | --- | --- | --- | --- | --- |
| Hewitt 2010  UK | Questionnaire | DM (CI) | Face to face interview and medical records | MMSE (≤23) | Self-report, glucose lowering medication or diagnosis in GP computerised records | NA | NA |  |
| Jara 2011  UK | Retrospective cohort | Dementia | Electronic medical records | Not specified |  |  | Cataract related surgical/diagnostic codes |  |
| Löppönen 2004  Finland | Cross-sectional | Dementia | Face to face interview and clinical exam | Diagnostic criteria for probable AD (NINADS-ADRDA)  DSM-IV – clinical exam | Diagnosis in medical records and/or history of stroke verified by clinical exam | Diagnosis in medical records and/or treatment with antidiabetic agents, and/or fasting plasma glucose level ≥ 7.0 mmol/l | NA |  |
| Lyketsos 2005  USA | Case-control | Dementia/CI | Face to face interview and clinical exam | Modified MMSE (3MS) or IQCODE (cognitive decline in elderly). Dementia questionnaire + clinical examination | Self or informant response | Self or informant response |  |  |
| McCormick 1994  USA | Case-control | Dementia/CI | Clinical exam and Medical records | Diagnostic criteria for probable AD (NINADS-ADRDA)  DSM-III-R (clinical exam) | Diagnosis in medical records | Diagnosis in medical records | Diagnosis in medical records | Charleston comorbidity index |
| Rait 2010  UK | Cohort | Dementia | Electronic medical records | Read codes | Read codes | Read codes |  |  |
| Sakurai 2010  Japan | Cross sectional | Dementia | Face to face interview and clinical exam | Diagnostic criteria for probable AD (NINADS-ADRDA) – clinical exam | Receiving diabetes therapy or over 5.9% in haemoglobin A1c or diagnosis (ADA guidelines) | NA | NA |  |
| **Study and country** | **Type of study** | **Type of population** | **Method of data collection** | **How dementia defined/assessed?** | **How diabetes defined/ recorded?** | **How stroke defined/recorded?** | **How VI defined/recorded?** | **Other** |
| Saposnik 2012  Canada | Retrospective cohort study | Stroke & dementia | Medical records and clinical exam | Any type of dementia recorded in notes | NA | Validated Canadian Neurological Scale (CNS) – clinical exam | NA |  |
| Schubert 2006  USA | Cross-sectional | Dementia | Medical records and clinical exam | ICD-10 - clinical exam | Coded ICD diagnosis | Coded ICD diagnosis | NA | Chronic disease Score |
| Stephan 2011  UK | Cross-sectional | MCI | Face to face interview | Mayo clinic criteria for MCI | Self or information response | Self or information response |  |  |
| Uhlmann 1991  USA | Case control | Dementia | Medical records | Diagnostic criteria for probable AD (NINADS-ADRDA) | NA | NA | Snellen & Rosenbaum methods |  |
| Whitson 2010  USA | Cross-sectional | VI (macular disease) | Clinical examination | TICS-m  WMS-R  Letter fluency (FAS) | NA | NA | Diagnosis in medical records |  |
| Yochim 2012 | Case series | VI (glaucoma) | Data collection face to face or via telephone | California verbal Learning Test (CVLT-II Short Form, Verbal Fluency test (D-KEFS) |  |  | Diagnosis in medical records |  |
| Zamrini 2004  USA | Case control | Dementia | Medical records | Diagnostic criteria for probable AD (NINADS-ADRDA) | Diagnosis in medical records | Diagnosis in medical records | Diagnosis in medical records |  |

| **Study and country** | **Type of study** | **Type of population** | **Method of data collection** | **How dementia defined/assessed?** | **How diabetes defined/ recorded?** | **How stroke defined/recorded?** | **How VI defined/recorded?** | **Other** |
| --- | --- | --- | --- | --- | --- | --- | --- | --- |
| Zekry 2008  Switzerland | Cohort | Dementia | Clinical exam and medical records | MMSE & short cognitive evaluation, diagnosis based on clinical criteria (clinical exam) | Diagnosis in medical records | Diagnosis in medical records | Diagnosis in medical records | Charleston comorbidity index |
| Zhang 2010  Australia | Retrospective cohort study | DM | Medical claims database | Database information on whether medication for dementia was dispensed. | ICD codes |  |  |  |
